# Salvage Autologous Stem Cell Transplantation in Relapsed/Refractory Multiple Myeloma: Long-Term Efficacy and Safety Outcomes from a Single-Center Real-World Cohort

**DOI:** 10.3390/jcm15187184

**Published:** 2026-09-16

**Authors:** Gulten Korkmaz, Funda Ceran, Simten Dagdas, Sertan Akgun, Kerim Kızılkaya, Ahmet Ceylan, Fahir Ozturk, Muruvvet Seda Aydın, Emel Isleyen, Merve Ecem Erdogan Yon, Gulsum Ozet

**Affiliations:** Department of Hematology, Ankara Bilkent City Hospital, Ankara 06800, Turkey; ceranf@gmail.com (F.C.); simtendagdas@gmail.com (S.D.); sertanakgun@gmail.com (S.A.); kerimkizilkaya@gmail.com (K.K.); ahmetceylanac@gmail.com (A.C.); drfahir@gmail.com (F.O.); drmseda84@gmail.com (M.S.A.); dremel.isleyen@gmail.com (E.I.); merveecemerdogan@hotmail.com (M.E.E.Y.); gulsumozet@gmail.com (G.O.)

**Keywords:** relapsed/refractory multiple myeloma, salvage autologous stem cell transplantation, progression-free survival, overall survival

## Abstract

**Background:** The role of salvage autologous stem cell transplantation (ASCT) in relapsed/refractory multiple myeloma (R/R MM) is being reconsidered in the era of novel immunotherapies. We evaluated the long-term efficacy and safety of salvage ASCT in a real-world single-center cohort. **Methods:** We retrospectively analyzed 57 patients with R/R MM who underwent salvage ASCT between March 2012 and August 2025. Clinical characteristics, treatment responses, toxicities, progression-free survival (PFS), overall survival (OS), and prognostic factors were assessed. **Results:** The mean age at salvage ASCT was 60.2 ± 8.2 years. Before salvage ASCT, 50.9% of patients achieved complete response or very good partial response, increasing to 80.7% by day +100. Transplant-related mortality was 3.5%, while infection or febrile neutropenia occurred in 77.2%. Median PFS and OS after salvage ASCT were 34.0 months (95% CI, 28.8–39.2) and 67.2 months (95% CI, 46.2–88.2), respectively. Median OS from diagnosis was 130.5 months. In multivariate analysis, higher LDH and pre-transplant refractory status were independently associated with PFS, whereas age at salvage ASCT and beta-2 microglobulin level were independently associated with OS. **Conclusions:** Salvage ASCT was associated with durable disease control and acceptable transplantation-related outcomes in this carefully selected cohort. Its comparative role relative to contemporary immunotherapies requires prospective evaluation.

## 1. Introduction

Multiple myeloma (MM) is a relatively rare plasma cell neoplasm, accounting for approximately 1–2% of all cancers [1,2]. Although survival outcomes have markedly improved with the introduction of proteasome inhibitors (PIs), immunomodulatory drugs (IMiDs), and anti-CD38 monoclonal antibodies, the disease continues to follow a relapsing course. High-dose chemotherapy followed by autologous stem cell transplantation (ASCT) was first shown to improve survival outcomes in MM in 1996 [3]. With the development of novel agents, it has been suggested that, particularly in standard-risk patients, stem cells may be collected and stored after 3–4 cycles of induction therapy and ASCT may be deferred. Nevertheless, high-dose melphalan-based consolidation supported by ASCT remains an important component of frontline therapy in eligible patients [4].

The introduction of bispecific antibodies and chimeric antigen receptor T-cell (CAR-T) therapies into clinical practice has substantially changed the treatment algorithm for relapsed/refractory multiple myeloma (R/R MM). In patients who relapse after a first ASCT, however, the role of a second or salvage ASCT is being reconsidered in the era of modern therapies [5,6,7,8,9]. Available evidence suggests that salvage ASCT should not be regarded as a standard approach for all patients with R/R MM but may provide clinical benefit in carefully selected patients, particularly those who achieved a prolonged remission following their first ASCT, have a good performance status, adequate organ function, and disease responsive to reinduction therapy [5,6,7,8]. In contrast, the long-term results of the GMMG ReLApsE trial demonstrated no significant progression-free survival (PFS) or overall survival (OS) advantage of salvage ASCT over continuous lenalidomide/dexamethasone therapy [9], further emphasizing the importance of appropriate patient selection.

In recent years, bispecific antibodies and CAR-T-cell therapies have substantially reshaped the treatment paradigm of R/R MM. The B-cell maturation antigen (BCMA)-targeting agents teclistamab and elranatamab, as well as the GPRC5D-targeting agent talquetamab, have demonstrated meaningful efficacy in heavily pretreated patients [10,11,12]. Similarly, high and deep response rates have been reported with the BCMA-directed CAR-T-cell therapies idecabtagene vicleucel (ide-cel) and ciltacabtagene autoleucel (cilta-cel) [13,14,15,16]. However, limitations related to accessibility, manufacturing, specialized center infrastructure, cost, and treatment-specific toxicities may restrict their use in routine clinical practice. Therefore, reassessing the clinical position of salvage ASCT in the era of modern immunotherapies using real-world data remains important.

In this study, we aimed to evaluate the long-term efficacy and safety outcomes of 57 patients who underwent salvage ASCT for R/R MM at our center and to define the potential role of salvage ASCT in the modern treatment era.

## 2. Materials and Methods

This retrospective study included patients who underwent salvage ASCT for R/R MM at our center between March 2012 and August 2025. Major guidelines, including those of the National Comprehensive Cancer Network, European Hematology Association/European Society for Medical Oncology, American Society for Transplantation and Cellular Therapy, European Society for Blood and Marrow Transplantation, and International Myeloma Working Group, recommend considering salvage ASCT in patients with a time to progression of at least 18 months after the first ASCT in the absence of maintenance therapy, or at least 36 months in patients who received maintenance therapy [17,18,19]. The duration of response following the first ASCT was defined as the interval from the date of the first ASCT to documented disease relapse or progression. Patients with R/R MM who relapsed after the first ASCT, whose duration of remission after the first ASCT was taken into consideration in accordance with guideline recommendations when assessing eligibility for salvage ASCT, who subsequently underwent salvage ASCT as part of salvage therapy, and for whom complete clinical and laboratory data were available were included in the study. Patients who underwent tandem autologous stem cell transplantation and those with incomplete medical records were excluded.

Data were obtained from patient files and electronic medical records and included age at diagnosis, age at salvage ASCT, sex, myeloma subtype, presence of extramedullary disease, International Staging System (ISS) stage at diagnosis, and Revised International Staging System (R-ISS) stage in patients with available cytogenetic data at diagnosis. Additional variables included response status before and after the first ASCT, induction regimen, number of induction cycles, maintenance therapy after the first ASCT, type of relapse (biochemical relapse/progression or aggressive clinical relapse), cytogenetic features and presence of extramedullary disease at relapse, response status and refractoriness before salvage ASCT, salvage ASCT-related toxicities according to the Common Terminology Criteria for Adverse Events version (CTCAE) 6.0, presence of engraftment failure, response at day 100 after salvage ASCT, maintenance therapy following salvage ASCT, development of subsequent relapse or secondary malignancy, and survival status at the last follow-up. Adverse events were graded according to CTCAE version 6.0 where sufficient documentation was available. Transplant-related mortality was defined as death from a transplantation-related cause within 100 days after salvage ASCT in the absence of disease progression.

Response assessment was performed according to the International Myeloma Working Group consensus criteria [20]. Progression-free survival (PFS) was defined as the time from salvage ASCT to disease progression, relapse, or death from any cause. Overall survival (OS) was calculated from the date of salvage ASCT to death from any cause or the date of last follow-up. In additional analyses, OS from the time of initial diagnosis to death or last follow-up was also evaluated. Follow-up information was obtained from outpatient records and electronic medical records. Patients were followed from the date of salvage ASCT until death or the last documented clinical assessment. Patients without an event were censored at the date of their last follow-up.

Ethical approval was obtained from the Ankara Bilkent City Hospital Ethics Committee on 24 September 2025 (approval no. TABED 1-25-1709).

### Statistical Analysis

Descriptive statistics were presented as mean ± standard deviation, median (minimum–maximum), or number and percentage, as appropriate. Missing data were not imputed. Analyses involving R-ISS and cytogenetic variables were performed as variable-specific complete-case analyses using only patients with available data. PFS and OS were estimated using the Kaplan–Meier method. The median follow-up duration was estimated using the reverse Kaplan–Meier method. The effects of potential prognostic factors on PFS and OS were evaluated using univariable and multivariable Cox proportional hazards regression analyses. Variables with *p* < 0.05 in the univariable analyses were considered candidates for multivariable analysis. The multivariable models were constructed using a forward stepwise likelihood-ratio (Forward LR) procedure, and only variables independently contributing to the model were retained. To address potential immortal-time bias, post-salvage ASCT maintenance therapy was modeled as a time-dependent covariate in an extended Cox proportional hazards model, with patients considered unexposed until the actual date of maintenance initiation and exposed thereafter. This model was adjusted for age at salvage ASCT and beta-2 microglobulin, which were baseline variables that remained independently associated with OS in the conventional multivariable analysis. The number of adjustment variables was limited because of the relatively small number of deaths and the potential risk of model overfitting. Statistical analyses were performed using IBM SPSS Statistics version 28.0 (IBM Corp., Armonk, NY, USA).

## 3. Results

A total of 57 patients with R/R MM who underwent salvage ASCT were included in the study. Of these, 53 were diagnosed between 2006 and 2017, and four between 2018 and 2022. The mean age at diagnosis was 53.8 ± 8.0 years, and the mean age at salvage ASCT was 60.2 ± 8.2 years; 56.1% of the patients were male. Extramedullary disease was present in 10.5% of patients at diagnosis and in 15.8% at relapse. Relapse was classified as clinical relapse in 29.8% of patients and biochemical relapse or progression in 70.2%. Among patients with available cytogenetic data at relapse, 11 (19.3%) had high-risk cytogenetic features. Before salvage ASCT, 93% of patients had an Eastern Cooperative Oncology Group performance status (ECOG PS) of 0–1. Detailed clinical and laboratory characteristics at diagnosis and relapse are presented in Table 1.

The most commonly used frontline induction regimens were bortezomib, cyclophosphamide, and dexamethasone (VCD) (35.1%) and vincristine, doxorubicin, and dexamethasone (VAD) (29.8%), reflecting the regimens reimbursed in our country during the years in which these patients were diagnosed. Before the first ASCT, 66.6% of patients had achieved a complete response (CR) or very good partial response (VGPR). No engraftment failure or transplant-related mortality (TRM) occurred following the first ASCT. At day 100 after transplantation, 94.7% of patients had achieved at least a VGPR. Maintenance therapy was administered to 15 patients (26.3%) after the first ASCT. The median time from the first ASCT to relapse or progression was 34 (18–154) months. The corresponding median durations were 48 months (36–108 months) among patients who received maintenance therapy after the first ASCT and 26.5 months (18–154 months) among those who did not. All 57 patients fulfilled the guideline-based interval criteria used for salvage ASCT eligibility: ≥18 months in the absence of maintenance therapy and ≥36 months among patients who received maintenance therapy after the first ASCT. Detailed treatment characteristics and outcomes related to the first ASCT are presented in Table 2.

The first ASCT procedures were performed between December 2006 and August 2022, and relapses after the first ASCT occurred between July 2009 and August 2025. During 2009–2014, relapse treatment consisted predominantly of VCD and VRD regimens. During 2015–2019, VCD and VRD remained commonly used, while carfilzomib-containing regimens became more frequent. During 2020–2025, carfilzomib-, pomalidomide-, and anti-CD38 antibody-based combinations were increasingly used. Salvage ASCT procedures were performed between March 2012 and August 2025, and progression following salvage ASCT occurred between November 2014 and March 2026. Treatments documented after progression in the earlier period mainly included VCD, VRD, RD, and KRD regimens. In later years, combinations containing carfilzomib, pomalidomide, ixazomib, selinexor, or anti-CD38 antibodies were increasingly used. Elranatamab also began to be used after it became available under national reimbursement in Türkiye in January 2025. Detailed post-progression treatment information was not consistently available for all patients.

After relapse, patients received a mean of 7.0 ± 3.5 cycles of therapy, and 75.4% had received only one line of treatment before salvage ASCT. Before salvage ASCT, 50.9% of patients were in CR or VGPR and 43.9% were in partial response (PR); 64.9% were not refractory to either a PI or an IMiD. Following salvage ASCT, the mean times to neutrophil and platelet engraftment were 11.0 ± 1.6 and 12.1 ± 4.3 days, respectively. Two patients (3.5%) received eltrombopag because of platelet engraftment failure. In 38.6% of patients, the melphalan dose was reduced to 140 mg/m^2^ because of impaired renal function or frailty. Two patients (3.5%) died from TRM after salvage ASCT; one death was due to sepsis and cardiac toxicity, and the other to SARS-CoV-2 pneumonia. Infection or febrile neutropenia was recorded as a composite outcome in 44 patients (77.2%). Non-hematologic toxicities were recorded in 14 patients (24.6%) and were predominantly grade 1–2 where grading information was available. At day 100 after salvage ASCT, 80.7% of patients had achieved CR or VGPR, whereas four patients (7.0%) had progressive disease. Maintenance therapy was initiated in 34 patients (59.6%) after salvage ASCT. During follow-up, 44 patients (77.2%) experienced relapse and six (10.5%) developed a secondary malignancy. These included bladder adenocarcinoma in two patients, colon adenocarcinoma in one, Philadelphia chromosome-positive B-cell acute lymphoblastic leukemia in one, cutaneous squamous cell carcinoma in one, and basal cell carcinoma in one. Except for the patient who developed basal cell carcinoma, all patients with secondary malignancies had also been exposed to an IMiD. At the last assessment, 23 patients (40.4%) were alive. The median follow-up duration after salvage ASCT, estimated using the reverse Kaplan–Meier method, was 78.0 months. Detailed treatment characteristics and follow-up outcomes related to salvage ASCT are presented in Table 3.

**Figure 1:**
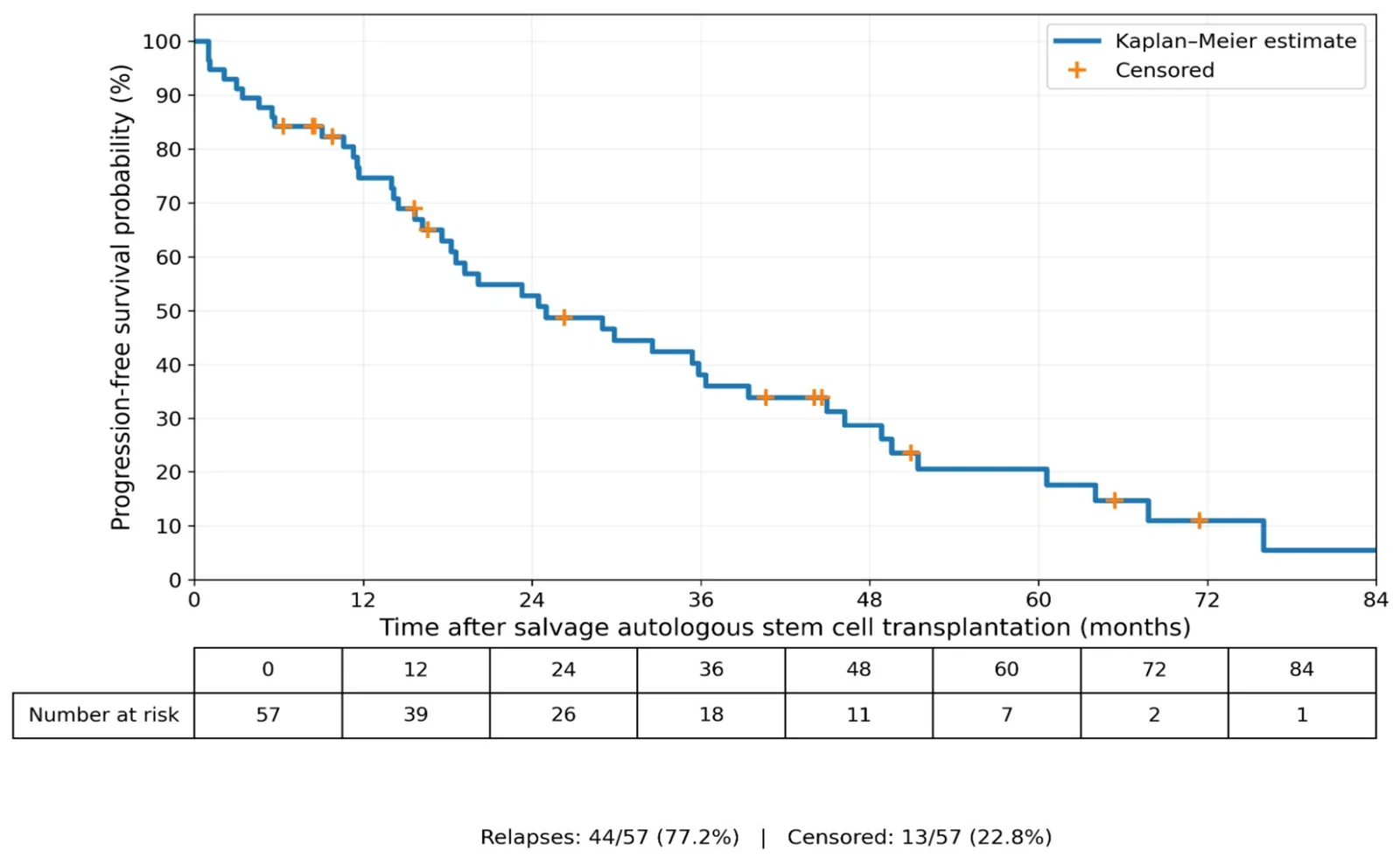
Progression-Free Survival (PFS) after salvage autologous stem cell transplantation.

**Figure 2:**
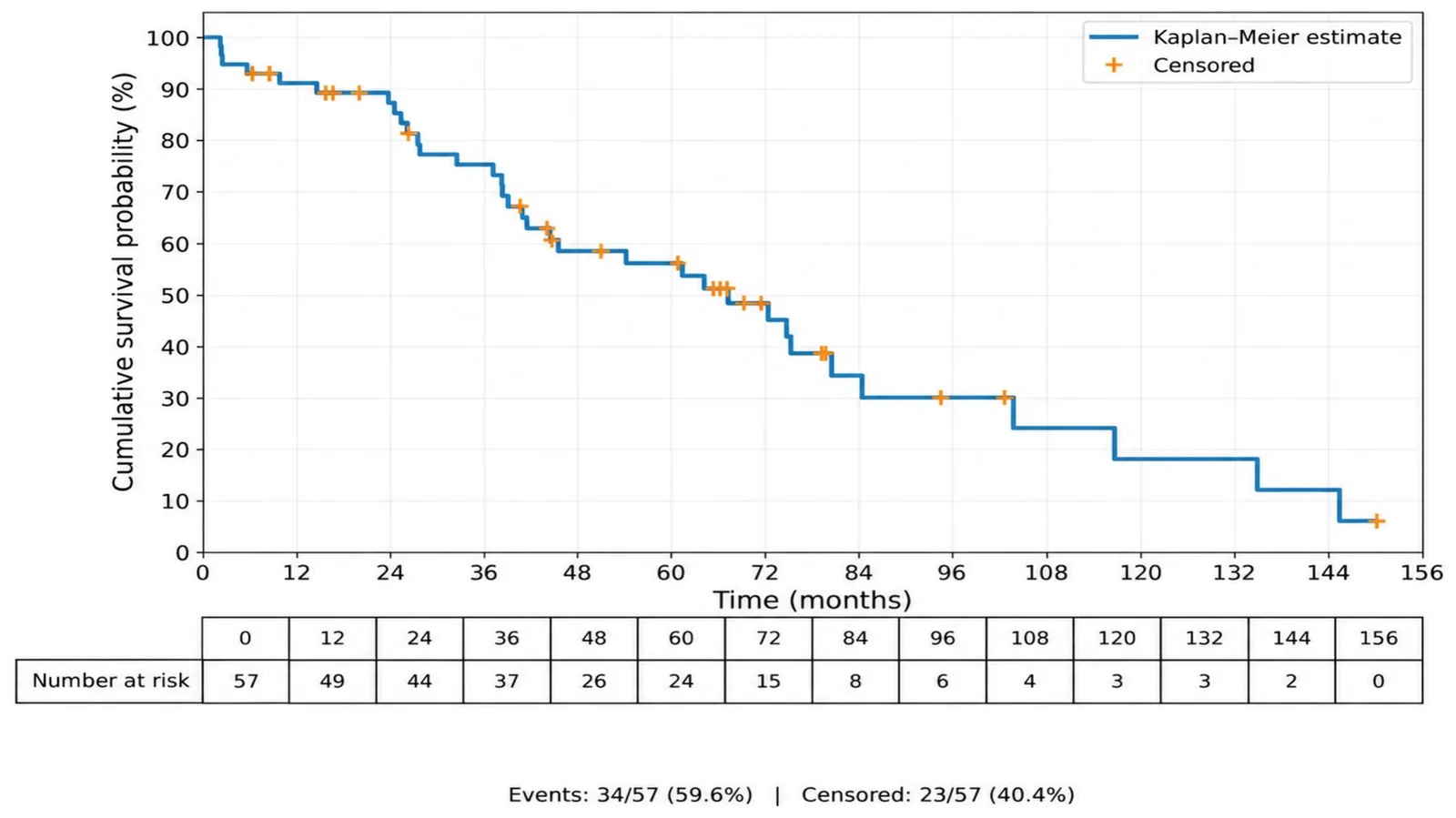
Overall Survival (OS) after salvage autologous stem cell transplantation.

**Figure 3:**
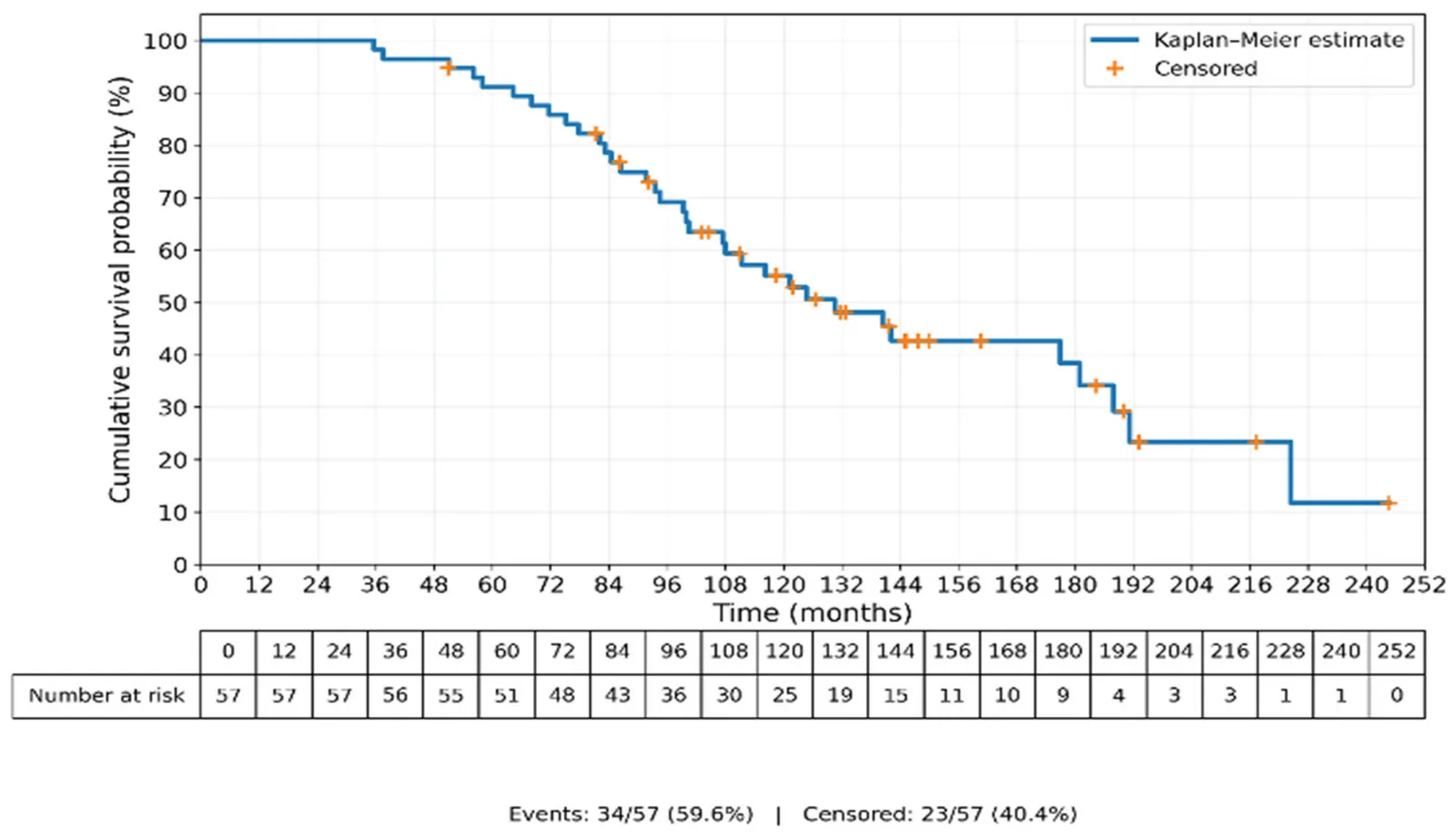
Overall Survival (OS) from the time of diagnosis.

Kaplan–Meier analysis showed a median PFS of 34.0 months (95% CI, 28.8–39.2) and a median OS of 67.2 months (95% CI, 46.2–88.2) following salvage ASCT. The median OS calculated from the time of initial diagnosis was 130.5 months (95% CI, 101.5–159.4) (Figure 1, Figure 2 and Figure 3).

The effects of various prognostic variables on PFS and OS were evaluated using univariate and multivariate Cox regression analyses. In the multivariate analysis, elevated LDH (HR, 1.007; 95% CI, 1.003–1.010; *p* = 0.001) and refractory status before salvage ASCT (HR, 1.414; 95% CI, 1.107–1.806; *p* = 0.005) were independently associated with PFS. In the conventional multivariable Cox analysis, age at salvage ASCT (HR, 1.053; 95% CI, 1.012–1.096; *p* = 0.011) and beta-2 microglobulin (HR, 1.137; 95% CI, 1.023–1.264; *p* = 0.017) remained independently associated with OS. To address potential immortal-time bias, maintenance therapy after salvage ASCT was evaluated separately as a time-dependent covariate. Among the 34 patients who received maintenance therapy, the median time from salvage ASCT to maintenance initiation was 96 (90–127) days. In the extended Cox model adjusted for age at salvage ASCT and beta-2 microglobulin, maintenance therapy was not significantly associated with OS (HR, 1.21; 95% CI, 0.56–2.61; *p* = 0.63). Detailed results of the univariate and multivariate analyses are presented in Table 4 and Table 5.

## 4. Discussion and Conclusions

In this single-center retrospective study, we evaluated the long-term outcomes of 57 patients with R/R MM who relapsed after a first ASCT and subsequently underwent salvage ASCT. The median PFS after salvage ASCT was 34 months and the median OS was 67.2 months, whereas the median OS from the time of initial diagnosis reached 130.5 months. Notably, while 50.9% of patients had achieved at least a VGPR before salvage ASCT, this proportion increased to 80.7% by day 100 after transplantation, indicating a substantial deepening of response following salvage ASCT. The TRM rate of 3.5% and the predominantly low-grade non-hematologic toxicities further suggest that salvage ASCT can provide meaningful and durable disease control in appropriately selected patients.

Our findings are generally consistent with recently published salvage ASCT series. In the single-center study by Bicsko et al. including 30 patients, the median PFS after a second ASCT was 24 months, the median OS was 48 months, and TRM was 3%. Furthermore, median PFS reached 32 months in patients who achieved CR or VGPR by day 100 after the second transplantation, compared with only 8.5 months in those who achieved PR [5]. In our cohort, the median PFS of 34 months and the approximately 81% rate of ≥VGPR at day 100 support the possibility of achieving prolonged disease control with salvage ASCT, particularly in treatment-sensitive patients. Similarly, in the KMM2301 study, the median PFS and OS among patients undergoing salvage ASCT were reported as 30 and 99 months, respectively [6]. Although the PFS observed in our study was comparable to these results, the shorter OS may be related to differences in patient characteristics, relapse biology, treatment approaches, and access to novel agents in subsequent lines of therapy. Indeed, 53 of the 57 patients in our cohort were diagnosed between 2006 and 2017, and our findings therefore largely reflect real-world practice before the widespread use of modern immunotherapies. In the study by Karp et al., the median PFS was 20.6 months, the median OS was 65 months, and 100-day mortality was 4%. Among low-risk patients with R-ISS stage I disease and a response duration of more than 24 months after the first transplant, median PFS and OS reached 45 and 80.2 months, respectively [7]. These findings indicate that the benefit derived from salvage ASCT is closely related to patient selection. Nevertheless, the role of salvage ASCT should be interpreted cautiously in light of prospective randomized data. In the long-term analysis of the GMMG ReLApsE trial, which compared salvage high-dose therapy and ASCT following lenalidomide/dexamethasone reinduction with continuous lenalidomide/dexamethasone therapy, median PFS was 20.5 versus 19.3 months and median OS was 67.1 versus 62.7 months, respectively, with no significant survival advantage demonstrated between the two approaches [9]. Therefore, rather than being routinely applied to all patients with relapsed disease, salvage ASCT appears more appropriately considered as an individualized treatment option in patients with favorable clinical and biological characteristics.

Over the past two decades, the treatment landscape of multiple myeloma has evolved substantially with the introduction of several novel agents, resulting in marked changes in the treatment regimens received by patients across different calendar periods. Consequently, our cohort was characterized by substantial temporal heterogeneity in treatment exposure. The increasing availability of carfilzomib-, pomalidomide-, anti-CD38 antibody-based, and, more recently, bispecific antibody therapies may have influenced OS independently of salvage ASCT. Because of the limited sample size, unequal follow-up across calendar periods, heterogeneous subsequent therapies, and incomplete post-progression treatment data, a reliable calendar-era survival analysis was not considered feasible. Therefore, OS should be interpreted in the context of evolving treatment availability and potential confounding by subsequent therapies.

The introduction of bispecific antibodies has substantially expanded the therapeutic options for R/R MM. In the MajesTEC-1 study, teclistamab achieved an overall response rate (ORR) of 63% and a median PFS of 11.3 months in heavily pretreated patients [10]. In the MagnetisMM-3 study, elranatamab achieved an ORR of 61%, demonstrating meaningful clinical activity [11]. The GPRC5D-targeting agent talquetamab also produced response rates exceeding 66% in heavily pretreated patients with R/R MM in the MonumenTAL-1 study [12]. However, the populations included in these studies differ substantially from our cohort. Patients enrolled in bispecific antibody trials had generally received multiple prior lines of therapy and had a high prevalence of refractory disease, whereas 75.4% of patients in our cohort proceeded to salvage ASCT after only one line of therapy following relapse, and the vast majority had achieved at least a partial response before transplantation. Rather than suggesting comparative superiority, these findings indicate that salvage ASCT may continue to provide clinically meaningful disease control in carefully selected, transplant-eligible patients treated relatively early in the relapse course. Although the “off-the-shelf” availability of bispecific antibodies represents an important advantage, the need for repeated or prolonged dosing and toxicities such as infections, cytopenias, hypogammaglobulinemia, and cytokine release syndrome (CRS) may impose a substantial treatment burden [11]. However, the populations included in these trials differed substantially from our cohort in terms of prior treatment exposure, refractory disease, and timing within the relapse course. Therefore, these results should be regarded only as contextual information and not as evidence of comparative efficacy or safety. In the absence of a comparator group, the relative effectiveness of salvage ASCT versus bispecific antibodies cannot be determined from our study.

CAR-T-cell therapies also provide high and deep response rates in R/R MM. The KarMMa trial demonstrated the efficacy of the BCMA-directed CAR-T-cell therapy ide-cel in heavily pretreated patients with R/R MM [13]. In the subsequent phase III KarMMa-3 trial, median PFS was 13.3 months with ide-cel compared with 4.4 months with standard regimens, while ORR was 71% and 42%, respectively [14]. In the CARTITUDE-1 study of cilta-cel, the ORR exceeded 97%, with 27-month PFS and OS rates of 54.9% and 70.4%, respectively [15,16]. However, because patients enrolled in these studies were generally treated at later lines of therapy and had a high prevalence of refractory disease, direct comparison with our salvage ASCT cohort would not be appropriate. These trial results are presented only to describe the evolving therapeutic landscape of R/R MM. Given the substantial differences in patient populations and the absence of a comparator group, our findings do not permit conclusions regarding the relative efficacy or safety of salvage ASCT compared with CAR-T-cell therapies.

In our study, the safety profile of salvage ASCT was acceptable. The TRM rate was 3.5%, comparable to the 3–4% rates reported in previous salvage ASCT series [5,7]. Although the melphalan dose was reduced to 140 mg/m^2^ in 38.6% of patients because of renal impairment or frailty, dose reduction was not significantly associated with survival. Infection or febrile neutropenia was common; however, non-hematologic toxicities occurred in 24.6% of patients and were predominantly grade 1–2 where grading information was available. Based on the available safety data, salvage ASCT appeared to have a manageable toxicity profile in appropriately selected patients; however, this finding should be interpreted cautiously because of the retrospective data collection and incomplete grading of adverse events.

Our prognostic analyses further support the importance of patient selection. In multivariate analysis, elevated LDH and refractory disease status before salvage ASCT were identified as independent adverse factors for PFS. These findings suggest that patients with a lower disease burden and treatment-sensitive disease may derive the greatest benefit from salvage ASCT. For OS, age at salvage ASCT and beta-2 microglobulin remained independently associated with outcome in the conventional multivariable analysis. However, when post-salvage ASCT maintenance therapy was modeled as a time-dependent covariate to address immortal-time bias, maintenance was not significantly associated with OS. Therefore, our findings do not demonstrate an independent OS benefit of maintenance therapy after salvage ASCT. This result should be interpreted cautiously because maintenance treatment was not randomized, the maintenance regimens were heterogeneous, and the relatively small number of patients and deaths limited statistical power. In particular, the association of parameters reflecting disease biology and treatment sensitivity with survival suggests that decisions regarding a second transplant should be based not only on the interval since the first ASCT, but also on disease characteristics at the time of relapse.

Our study has several limitations. Its retrospective, single-center design and limited sample size reduce the statistical power of subgroup and multivariate analyses. Despite using parsimonious models, model instability and overfitting cannot be excluded because of the limited sample size and number of events. Formal collinearity diagnostics were not performed, and the proportional-hazards assumption was not formally tested. Therefore, the multivariable findings should be interpreted as exploratory. Owing to the retrospective design, detailed infection-specific and microbiological data were not consistently available for all patients. Therefore, infection and febrile neutropenia could not be reliably analyzed as separate outcomes, and the overall frequency of grade ≥ 3 adverse events could not be determined accurately. In addition, because the majority of the cohort was diagnosed between 2006 and 2017, frontline and relapse treatments do not fully reflect current standards of care. The absence of R-ISS and cytogenetic data at diagnosis in a substantial proportion of patients also limited comprehensive biological risk assessment. Moreover, the non-random availability of cytogenetic testing may have introduced selection bias. The hazard-ratio estimates for high-risk cytogenetics at diagnosis should be interpreted cautiously because this feature was present in only six patients, resulting in imprecise estimates and wide confidence intervals. Direct comparison between salvage ASCT and contemporary therapies was not possible because anti-CD38 antibodies and bispecific antibody therapies were not widely available during much of the study period, while CAR-T-cell therapy remains unavailable in our country. Furthermore, because the study included no comparator group and did not perform formal comparative-effectiveness or economic analyses, no conclusions can be drawn regarding the relative efficacy, accessibility, or cost-effectiveness of salvage ASCT compared with contemporary immunotherapies. Nevertheless, the long follow-up duration, detailed transplantation-related outcomes, availability of both PFS and OS analyses, and demonstration of real-world outcomes achievable with salvage ASCT before the modern immunotherapy era represent important strengths of this study.

In conclusion, our findings suggest that salvage ASCT may provide meaningful disease control in carefully selected patients with R/R MM, particularly those with prolonged remission after the first ASCT, treatment-sensitive relapse, good performance status, and adequate stem cell reserve. However, because this was an uncontrolled retrospective study without comparative-effectiveness or economic analyses, our findings do not establish the superiority, relative accessibility, or economic advantage of salvage ASCT over contemporary immunotherapies. Salvage ASCT should not be regarded as a replacement for modern immunotherapies but may be considered as part of an individualized treatment-sequencing strategy. Prospective comparative studies are required to define its optimal position in the era of CAR-T-cell and bispecific-antibody therapies.

## Figures and Tables

**Table 1:** Patient characteristics at diagnosis and at the time of first and salvage autologous stem cell transplantation.

Variable	Category	Min–Max	Mean ± SD	*n* (%)
Age at diagnosis, years		34.0–66.0	53.8 ± 8.0	
Age at salvage ASCT, years		39.0–73.0	60.2 ± 8.2	
Sex	Female			25 (43.9%)
	Male			32 (56.1%)
Heavy- and light-chain isotype	IgG kappa			18 (31.6%)
	IgG lambda			16 (28.1%)
	IgA kappa			6 (10.5%)
	IgA lambda			5 (8.8%)
	Kappa light chain			6 (10.5%)
	Lambda light chain			6 (10.5%)
Extramedullary disease at diagnosis	Yes			6 (10.5%)
	No			51 (89.5%)
ISS stage	I			19 (33.3%)
	II			26 (45.6%)
	III			12 (21.1%)
R-ISS stage	I			8 (14.0%)
	II			12 (21.1%)
	III			4 (7.0%)
	Not available			33 (57.9%)
LDH	Normal			37 (64.9%)
	Elevated			20 (35.1%)
Beta-2 microglobulin, mg/L		1.6–16.3	4.3 ± 2.7	
Creatinine, mg/dL		0.7–9.4	2.0 ± 1.6	
Calcium, mg/dL		8.4–14.6	10.1 ± 1.4	
Hemoglobin, g/dL		7.0–15.0	9.8 ± 1.7	
Cytogenetic risk at diagnosis *	Standard risk			18 (31.6%)
	High risk **			6 (10.5%)
	Not available			33 (57.9%)
Type of relapse	Clinical relapse			17 (29.8%)
	Biochemical relapse or progression			40 (70.2%)
Cytogenetic risk at relapse *	Standard risk			31 (54.4%)
	High risk			11 (19.3%)
	Not available			15 (26.3%)
Extramedullary disease at relapse	Yes			9 (15.8%)
	No			48 (84.2%)
ECOG PS before salvage ASCT	0			41 (71.9%)
	1			12 (21.1%)
	2			4 (7.0%)

ASCT: autologous stem cell transplantation; ECOG PS: Eastern Cooperative Oncology Group Performance Status; ISS: International Staging System; LDH: lactate dehydrogenase; R-ISS: Revised International Staging System; * Cytogenetic data were not available for all patients; ** High-risk cytogenetic abnormalities were defined as t(4; 14), t(14; 16), del(17p13), and gain(1q21).

**Table 2:** Treatment characteristics and clinical outcomes of the first autologous stem cell transplantations.

Variable	Category	Min–Max	Mean ± SD	*n* (%)
Induction regimen	VCD			20 (35.1%)
	VAD			17 (29.8%)
	VAD + VCD			14 (24.6%)
	VCD + VRD			4 (7.0%)
	VCD + VDT-PACE			2 (3.5%)
Number of induction cycles		3.0–8.0	4.7 ± 1.5	
Number of treatment lines before the first ASCT	1			40 (70.2%)
	2			17 (29.8%)
Response before the first ASCT	CR			19 (33.3%)
	PR			18 (31.6%)
	VGPR			19 (33.3%)
	SD			1 (1.8%)
Neutrophil engraftment after the first ASCT, days		8.0–17.0	10.6 ± 1.6	
Platelet engraftment after the first ASCT, days		8.0–21.0	11.1 ± 2.4	
Engraftment failure after the first ASCT, days	No			57 (100.0%)
TRM after first ASCT	No			57 (100.0%)
Day +100 response after the first ASCT	CR			33 (57.9%)
	PR			3 (5.3%)
	VGPR			21 (36.8%)
Maintenance therapy after the first ASCT	No			42 (73.7%)
	Yes			15 (26.3%)
Type of maintenance therapy after the first ASCT *	Lenalidomide			8 (53.3%)
	Thalidomide			6 (40.0%)
	Bortezomib			1 (6.7%)
Time from first ASCT to relapse/progression, months **	Overall	18–154	34 (21–52)	
	Maintenance after first ASCT	36–108	48 (38.5–60)	
	No maintenance after first ASCT	18–154	26.5 (19.25–45)	

ASCT: autologous stem cell transplantation; CR: complete response; PR: partial response; SD: stable disease; TRM, transplant-related mortality; VAD: vincristine, doxorubicin, and dexamethasone; VCD: bortezomib, cyclophosphamide, and dexamethasone; VDT-PACE: bortezomib, dexamethasone, thalidomide, cisplatin, doxorubicin, cyclophosphamide, and etoposide; VGPR: very good partial response; VRD: bortezomib, lenalidomide, and dexamethasone; * Percentages were calculated among the 15 patients who received maintenance therapy after the first ASCT; ** Defined as the interval from the date of the first ASCT to documented relapse or disease progression.

**Table 3:** Treatment characteristics and clinical outcomes of the salvage autologous stem cell transplantations.

Variable	Category	Min–Max	Mean ± SD	*n* (%)
Number of treatment cycles after relapse		2.0–16.0	7.0 ± 3.5	
Number of treatment lines after relapse	1			43 (75.4%)
	2			9 (15.8%)
	3			3 (5.3%)
	4			2 (3.5%)
Response before salvage ASCT	CR			14 (24.6%)
	PR			25 (43.9%)
	VGPR			15 (26.3%)
	SD			3 (5.3%)
Refractory status before salvage ASCT	Non-refractory			37 (64.9%)
	PI-refractory			6 (10.5%)
	IMiD-refractory			5 (8.8%)
	PI- and IMiD-refractory			9 (15.8%)
Neutrophil engraftment after salvage ASCT, days		9.0–18.0	11.0 ± 1.6	
Platelet engraftment after salvage ASCT, days		7.0–34.0	12.1 ± 4.3	
Engraftment failure after salvage ASCT	Yes			2 (3.5%)
	No			55 (96.5%)
Melphalan dose reduction	Yes			22 (38.6%)
	No			35 (61.4%)
TRM after salvage ASCT	No			55 (96.5%)
	Yes			2 (3.5%)
Infection or febrile neutropenia	No			13 (22.8%)
	Yes			44 (77.2%)
Non-hematologic toxicity	No			43 (75.4%)
	Yes			14 (24.6%)
Day +100 response after salvage ASCT	CR			29 (50.9%)
	PR			7 (12.3%)
	VGPR			17 (29.8%)
	Progressive disease			4 (7.0%)
Maintenance therapy after salvage ASCT	No *			23 (40.4%)
	Yes			34 (59.6%)
Type of maintenance therapy after salvage ASCT **	Lenalidomide			16 (47.1%)
	Bortezomib			8 (23.5%)
	Bortezomib + lenalidomide			3 (8.8%)
	Daratumumab + bortezomib			4 (11.8%)
	Carfilzomib			3 (8.8%)
Relapse after salvage ASCT	Yes			44 (77.2%)
	No			13 (22.8%)
Secondary malignancy	Yes			6 (10.5%)
	No			51 (89.5%)
Disease status at the last follow-up	CR			14 (24.6%)
	PR			1 (1,7%)
	VGPR			3 (5.3%)
	Progressive disease			39 (68.4%)
Mortality status	Deceased			34 (59.6%)
	Alive			23 (40.4%)

ASCT: autologous stem cell transplantation; CR: complete response; IMiD: immunomodulatory drug; PI: proteasome inhibitor; PR: partial response; SD: stable disease; TRM: transplant-related mortality; * Maintenance therapy was not initiated in four patients because treatment was started for disease progression; ** Percentages were calculated among the 34 patients who received maintenance therapy after salvage ASCT.

**Table 4:** Univariate and multivariate Cox regression analyses of factors associated with progression-free survival after salvage autologous stem cell transplantation.

Variable	Univariate Cox Analysis					Multivariate Cox Analysis				
	HR	95% CI			*p*	HR	95% CI			*p*
ISS stage ^†^	1.776	1.243	-	2.538	0.002					
R-ISS stage *^,†^	2.179	1.042	-	4.559	0.039					
LDH (U/L)	1.006	1.003	-	1.010	0.001	1.007	1.003	-	1.010	0.001
Beta-2 microglobulin (mg/L)	1.134	1.044	-	1.232	0.003					
Creatinine (mg/dL)	1.006	0.846	-	1.195	0.948					
Calcium (mg/dL)	1.209	1.009	-	1.448	0.040					
Hemoglobin (g/dL)	0.826	0.694	-	0.982	0.030					
Cytogenetic risk at diagnosis *^,††^	17.28	3.332	-	89.64	0.001					
Response before the first ASCT (PR/SD vs. CR/VGPR)	1.365	1.003	-	1.857	0.048					
Neutrophil engraftment after the first ASCT, days	1.182	1.012	-	1.381	0.034					
Platelet engraftment after the first ASCT, days	1.011	0.906	-	1.129	0.844					
Maintenance therapy after the first ASCT (Yes vs. No)	0.310	0.122	-	0.788	0.014					
Cytogenetic risk at relapse *^,††^	1.888	0.932	-	3.822	0.077					
Number of treatment lines after relapse	1.451	1.009	-	2.086	0.045					
Response before salvage ASCT (PR/SD vs. CR/VGPR)	0.867	0.693	-	1.085	0.212					
Refractory status before salvage ASCT **	1.385	1.086	-	1.767	0.009	1.414	1.107	-	1.806	0.005
Maintenance therapy after salvage ASCT (Yes vs. No)	0.983	0.951	-	1.016	0.306					

ISS: International Staging System; R-ISS: Revised International Staging System. ^†^ ISS and R-ISS stages were analyzed as ordinal variables; HRs represent the effect of a one-stage increase; * Analyses were restricted to patients with available data. Analyses involving R-ISS and cytogenetic risk at diagnosis included 24 patients, with 16 PFS events; analyses involving cytogenetic risk at relapse included 42 patients, with 30 PFS events; LDH: lactate dehydrogenase; ASCT: autologous stem cell transplantation; ^††^ Cytogenetic risk was analyzed as high-risk versus standard-risk, high-risk cytogenetic abnormalities were defined as t(4; 14), t(14; 16), del(17p13), and gain(1q21); PR: partial response; SD: stable disease; CR: complete response; VGPR: very good partial response; ** Refractory status before salvage ASCT was analyzed as a binary variable: refractory vs. non-refractory, the refractory group included patients with PI (proteasome inhibitor)-refractory, IMiD (immunomodulatory drug)-refractory, or dual PI/IMiD-refractory disease.

**Table 5:** Univariate and multivariate Cox regression analyses of factors associated with overall survival after salvage autologous stem cell transplantation.

Variable	Univariate Cox Analysis					Multivariate Cox Analysis				
	HR	95% CI			*p*	HR	95% CI			*p*
Age at salvage ASCT (years)	1.050	1.010	-	1.092	0.014	1.053	1.012	-	1.096	0.011
ISS stage ^†^	2.249	1.339	-	3.780	0.002					
R-ISS stage *^,†^	1.205	0.505	-	2.879	0.674					
LDH (U/L)	1.009	1.004	-	1.014	0.002					
Beta-2 microglobulin (mg/L)	1.224	1.111	-	1.349	0.001	1.137	1.023	-	1.264	0.017
Creatinine (mg/dL)	1.101	0.902	-	1.343	0.344					
Calcium (mg/dL)	1.186	0.895	-	1.571	0.235					
Hemoglobin (g/dL)	0.815	0.648	-	1.024	0.078					
Cytogenetic risk at diagnosis *^,††^	10.02	1.654	-	60.75	0.012					
Response before the first ASCT (PR/SD vs. CR/VGPR)	1.514	1.001	-	2.290	0.050					
Cytogenetic risk at relapse *^,††^	3.408	1.337	-	8.685	0.010					
Number of treatment lines after relapse	2.125	1.255	-	3.595	0.005					
Response before salvage ASCT (PR/SD vs. CR/VGPR)	1.088	0.832	-	1.423	0.536					
Refractory status before salvage ASCT **	1.522	1.095	-	2.116	0.012					
Neutrophil engraftment after salvage ASCT, days	1.402	1.070	-	1.837	0.014					
Platelet engraftment after salvage ASCT, days	1.036	0.963	-	1.114	0.342					
Maintenance therapy after salvage ASCT ***						1.21	0.56	-	2.61	0.63

ASCT: autologous stem cell transplantation; ISS: International Staging System; R-ISS: Revised International Staging System; ^†^ ISS and R-ISS stages were analyzed as ordinal variables; HRs represent the effect of a one-stage increase; * Analyses were restricted to patients with available data. Analyses involving R-ISS and cytogenetic risk at diagnosis included 24 patients, with 10 deaths; analyses involving cytogenetic risk at relapse included 42 patients, with 20 deaths; LDH: lactate dehydrogenase; ^††^ Cytogenetic risk was analyzed as high-risk versus standard-risk, high-risk cytogenetic abnormalities were defined as t(4; 14), t(14; 16), del(17p13), and gain(1q21); PR: partial response; SD: stable disease; CR: complete response; VGPR: very good partial response; ** Refractory status before salvage ASCT was analyzed as a binary variable: refractory vs. non-refractory, the refractory group included patients with PI (proteasome inhibitor)-refractory, IMiD (immunomodulatory drug)-refractory, or dual PI/IMiD-refractory disease; *** Maintenance therapy after salvage ASCT was analyzed separately as a time-dependent covariate to account for immortal-time bias. The model was adjusted for age at salvage ASCT and beta-2 microglobulin.

## Data Availability

The original contributions presented in this study are included in the article. Further inquiries can be directed to the corresponding author.
